# Co-localization of lymphoid aggregates and lymphatic networks in nose- (NALT) and lacrimal duct-associated lymphoid tissue (LDALT) of mice

**DOI:** 10.1186/s12865-018-0242-3

**Published:** 2018-01-25

**Authors:** Melanie Lohrberg, Reinhard Pabst, Jörg Wilting

**Affiliations:** 1 Institute for Anatomy and Cell Biology, University Medical Hospital Göttingen, Kreuzbergring 36, D-37075 Göttingen, Germany; 2 Institute for Immunomorphology, Medical School Hannover, Carl-Neuberg-Str. 1, Hannover, D-30625 Germany; 3 Institute for Neuropathology, University Medical Hospital Göttingen, Robert-Koch-Strasse 40, D-37075 Göttingen, Germany

**Keywords:** Lymphatic vessels, Lymphatic endothelial cell, Lymphoid tissue, Mouse head, NALT, LDALT, Lyve-1, Podoplanin, Mucosal immunity

## Abstract

**Background:**

The lymphatic vascular pattern in the head of mice has rarely been studied, due to problems of sectioning and immunostaining of complex bony structures. Therefore, the association of head lymphoid tissues with the lymphatics has remained unknown although the mouse is the most often used species in immunology.

**Results:**

Here, we studied the association of nasal and nasolacrimal duct lymphatics with lymphoid aggregates in 14-day-old and 2-month-old mice. We performed paraffin sectioning of whole, decalcified heads, and immunostaining with the lymphatic endothelial cell-specific antibodies Lyve-1 and Podoplanin. Most parts of the nasal mucous membrane do not contain any lymphatics. Only the region of the inferior turbinates contains lymphatic networks, which are connected to those of the palatine. Nose-associated lymphoid tissue (NALT) is restricted to the basal parts of the nose, which contain lymphatics. NALT is continued occipitally and can be found at both sides along the sphenoidal sinus, again in close association with lymphatic networks. Nasal lymphatics are connected to those of the ocular region via a lymphatic network along the nasolacrimal duct (NLD). By this means, lacrimal duct-associated lymphoid tissue (LDALT) has a dense supply with lymphatics.

**Conclusions:**

NALT and LDALT play a key role in the immune system of the mouse head, where they function as primary recognition sites for antigens. Using the dense lymphatic networks along the NLD described in this study, these antigens reach lymphatics near the palatine and are further drained to lymph nodes of the head and neck region.

NALT and LDALT develop in immediate vicinity of lymphatic vessels. Therefore, we suggest a causative connection of lymphatic vessels and the development of lymphoid tissues.

## Background

Cells of the immune system are present in almost all tissues of the body, with highest density in primary and secondary lymphatic organs. It is known that lymphatic vessels are part of the immune response by providing routes for the delivery of antigens and antigen presenting cells to the draining lymph nodes. For their circulation through the body, immune cells use afferent routes, usually the blood and lymph vessels and specialized high endothelial venules (HEVs), as well as efferent routes, usually the lymphatic vessels. Therefore, secondary lymphatic organs possess either only efferent lymphatics (spleen, tonsils, Peyer’s patches) or both afferent and efferent lymphatics (lymph nodes), whereas the primary lymphatic organ the bone marrow, does not possess lymphatics. In contrast, the other primary lymphoid organ, the thymus, has efferent lymphatics [1]. Much of the lymphoid tissue in the body is located in the skin and near mucous membranes, accordingly named skin- and mucosa-associated lymphoid tissue (SALT, MALT). MALT can predominantly be found in the gastro-intestinal tract (gut-associated lymphoid tissue, GALT) and respiratory tract (bronchus-associated lymphoid tissue, BALT, [2]), where they induce mucosal immune responses. The basic structure and species differences of the respiratory tract in respect to immune reaction has recently been summarized [3].

In the human head, most of the lymphoid tissue is organized as tonsils in Waldeyer’s ring, whereas rodents do not have tonsils. It has been described for various species, including humans and rodents, that a functionally important proportion of the lymphoid tissue of the head can be found in the nasal passages, forming the nose-associated lymphoid tissue (NALT) [4–9]. In rodents, NALT is localized frontally of the soft palatine, where it forms a non-encapsulated lymphoid aggregate composed of T- and B-cells, HEVs and dendritic cells (DCs), covered by an epithelium with rare goblet cells and M-cells [5, 10, 11]. In contrast, human NALT was only observed in approximately 40% of investigated children, and is not located at a defined site, but disseminated throughout different parts of the nose [9]. A still largely unknown proportion of lymphoid tissue of the head is situated in the vicinity of the lacrimal duct, it can be found in humans and rodents and is named lacrimal duct-associated lymphoid tissue (LDALT). In rodents, this lymphoid tissue is also often referred to as tear duct-associated lymphoid tissue (TALT) [12–15].

In contrast to most secondary lymphatic organs, the initiation of development of NALT and LDALT starts after birth. In mice, the development of NALT is known to be completed 6 weeks after birth, even though its structure still undergoes substantial changes later on, probably reacting to environmental stimuli [16–18]. It is known that the cellular and molecular requirements for the formation of the different mucosa-associated lymphoid tissues vary (for review see: [19]), but it is widely accepted that the development of most secondary lymphoid organs relies on a crossplay between stromal organizer cells and CD45^+^CD3^−^CD4^+^ lymphoid tissue inducer (LTi) cells. For murine NALT development, signaling cascades including the chemokine receptor CXCR5 and the lymphotoxin β receptor (LTβR) initiate the formation of HEVs and thereby allow the recruitment of lymphocytes [20]. Although the distinct molecular mechanisms of murine NALT and LDALT development remain to be clarified, it is known that its development can occur independently of several factors necessary for the development of e.g. lymph nodes or Peyer’s patches [16–18, 21–24]. Recent publications are suggesting a causative connection between the presence of lymphatic vessels and lymphoid tissue formation. For example, Onder and coworkers showed that lymphatic endothelial cells are able to control the retention of LTi cells in lymph node anlagen of embryonic mice by cell-intrinsic RANK-signaling. Thereby, LECs play a key role in lymph node development [25]. Moreover, it was described that lymphoid aggregates forming in the bowel of Crohn’s disease patients are in close contact to efferent and afferent lymph vessels of nearby lymph nodes, with B-cells and innate lymphoid cells invading the vessel wall of these lymphatics, possibly mediating lymphatic remodeling [26].

In a previous study, we investigated the lymphatic networks in the head of 14-day-old mice and observed that the overwhelming proportion of the mucous membrane of the nose is devoid of lymphatics. However, in the most basal parts of the nasal mucous membrane we found lymphatic vessels that were connected to those of the ocular region by a lymphatic network that accompanies the nasolacrimal duct (NLD). Via this route, interstitial fluid of the ocular region is drained into nasal and palatinal lymphatics, and, via pharyngeal lymphatics, reaches the deep cervical lymph nodes [27]. Following up on this study, we investigated here the location of lymphatic vessels in the regions of the main mucosa-associated lymphoid tissues of the mouse head, NALT and LDALT. Thereby, we describe the distinct route of antigens from primary entry sites at mucous membranes to cervical lymph nodes, leading to mucosal immunity. Moreover, we investigated the presence of lymphatic vessels in close proximity to NALT and LDALT, supporting the hypothesis that the development of lymphoid tissues is directly connected to the presence of lymphatic vessels. As it is known that the development of murine NALT is finished 6 weeks after birth, we investigated 14-day-old and 2-month-old mice. Thereby, we depict on the one hand developing lymphoid tissue (14-day-old mice) and on the other hand mature lymphoid tissue (2-month-old mice).

## Results

**Fig. 1:**
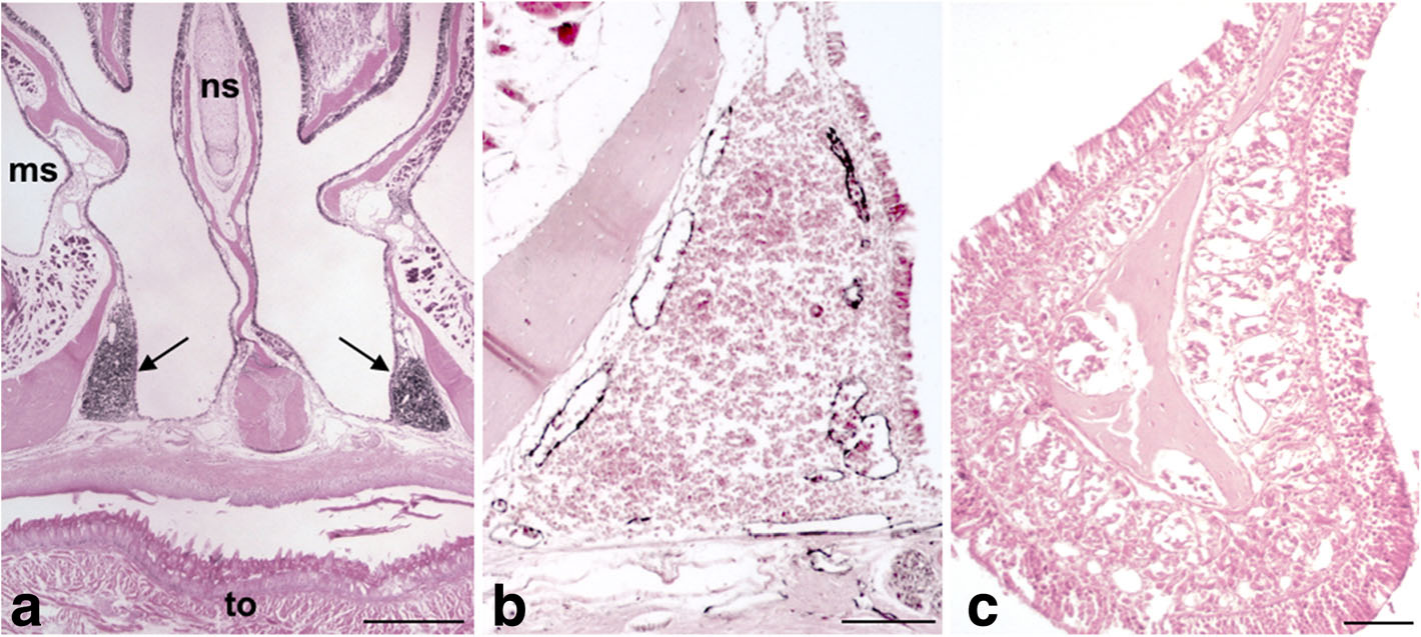
Mature NALT and adjacent lymphatics in 2-month-old mice. **a** HE-staining showing the nasal cavity and the position of NALT (arrows) in the inferior turbinates adjacent to the palatine. (ms, maxillary sinus; ns, nasal septum; to, tongue) 20× magnification, scale bar represents 500 μm. **b**, **c** Tyramide-enhanced anti-Lyve-1 immunoperoxidase staining showing numerous lymphatics (black) in NALT (**b**), and absence of lymphatics in most other parts of the nasal mucous membrane (**c**). 100× magnification, scale bar represents 100 μm

**Fig. 2:**
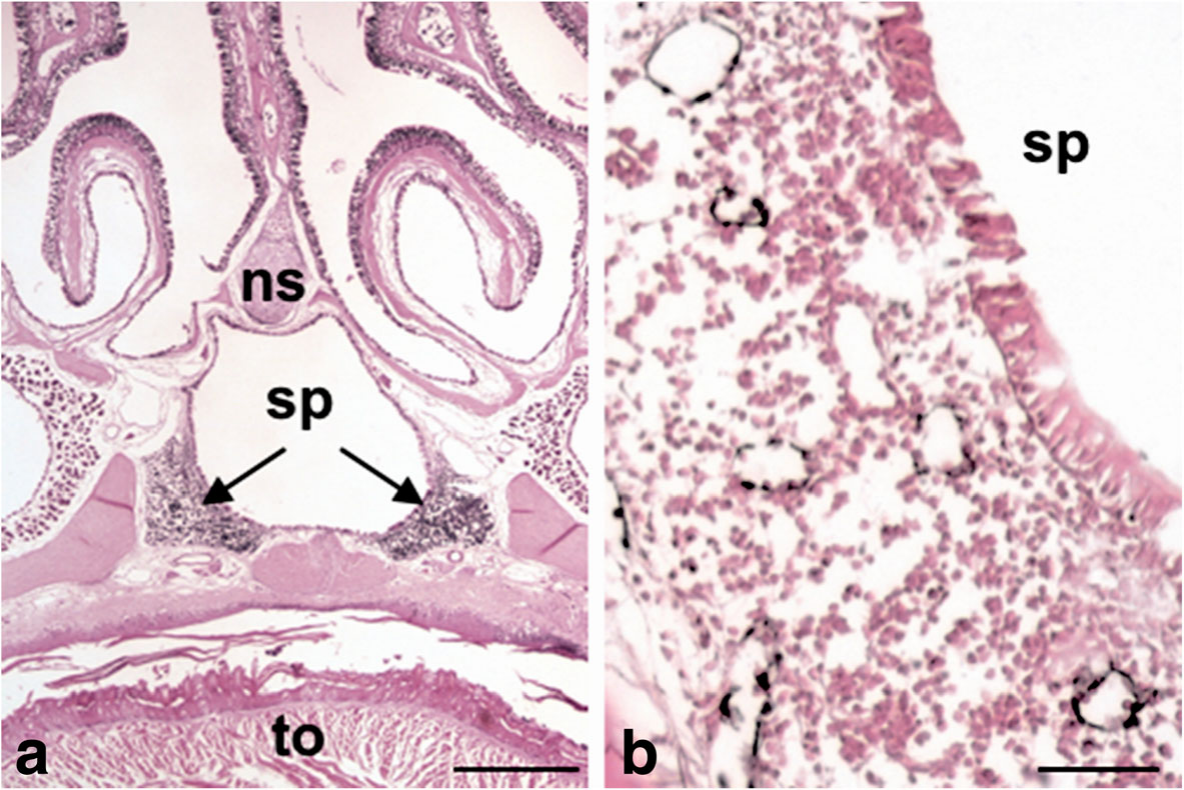
Lymphoid tissue near the sphenoidal sinus and adjacent lymphatics in 2-month-old mice. **a** Overview showing the region of the sphenoidal sinus (sp) with aggregates of lymphoid tissue (arrows) (ns, nasal septum). 20× magnification, scale bar represents 500 μm. **b** Tyramide-enhanced anti-Lyve-1 immunoperoxidase staining showing numerous lymphatics (black) in the sinus-associated lymphoid tissue. 200× magnification, scale bar represents 50 μm

**Fig. 3:**
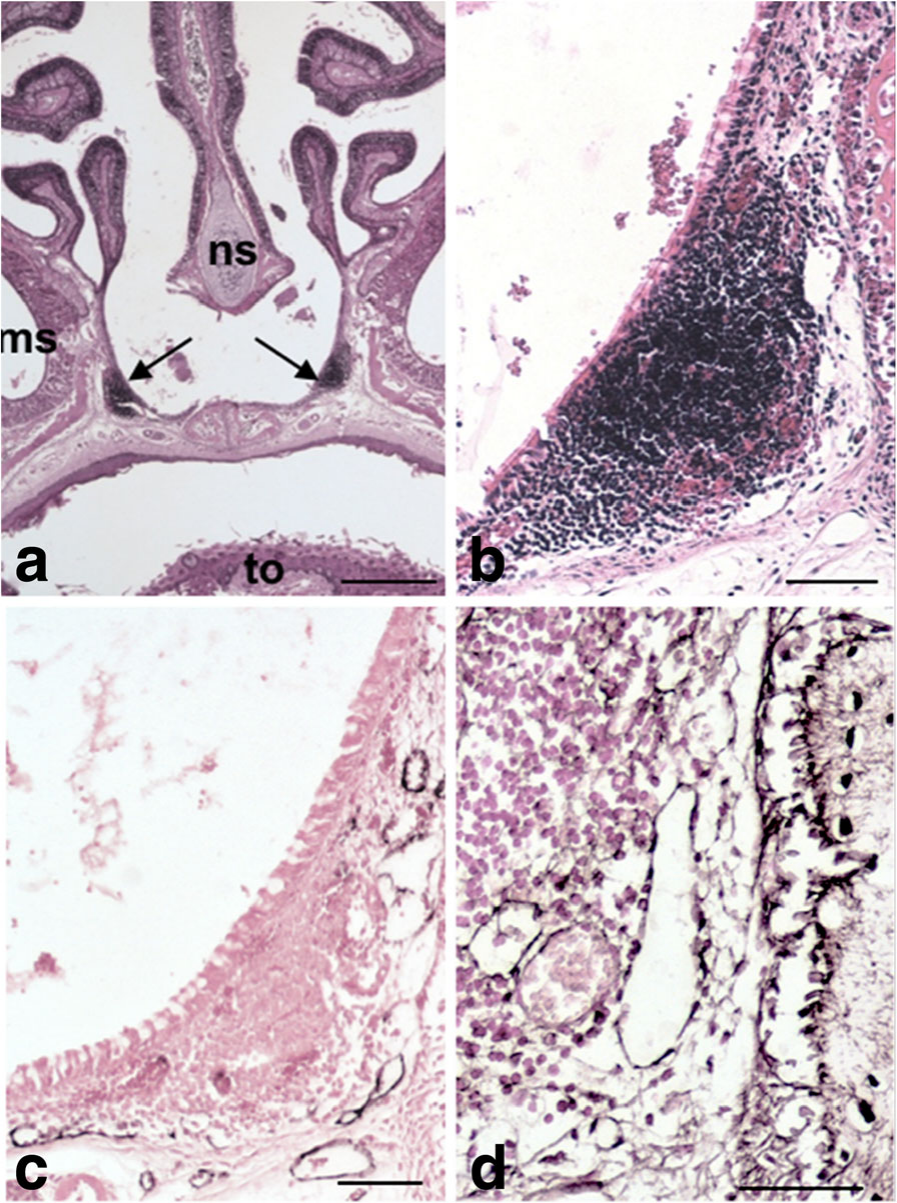
Developing NALT and adjacent lymphatics in 14-day-old mice. **a, b** HE-staining showing the nasal cavity and the position of NALT (arrows). 20× magnification, scale bar represents 500 μm (**a**); 100× magnification, scale bar represents 100 μm. **c** Anti-Lyve-1 immunoperoxidase staining showing lymphatics near the NALT. 100× magnification, scale bar represents 100 μm. **d** Tyramide-enhanced anti-podoplanin immunoperoxidase staining showing lymphatics and other podoplanin-positive structures in the NALT-region. 200× magnification, scale bar represents 50 μm. Other podoplanin-positive structures are indicated in the results part

**Fig. 4:**
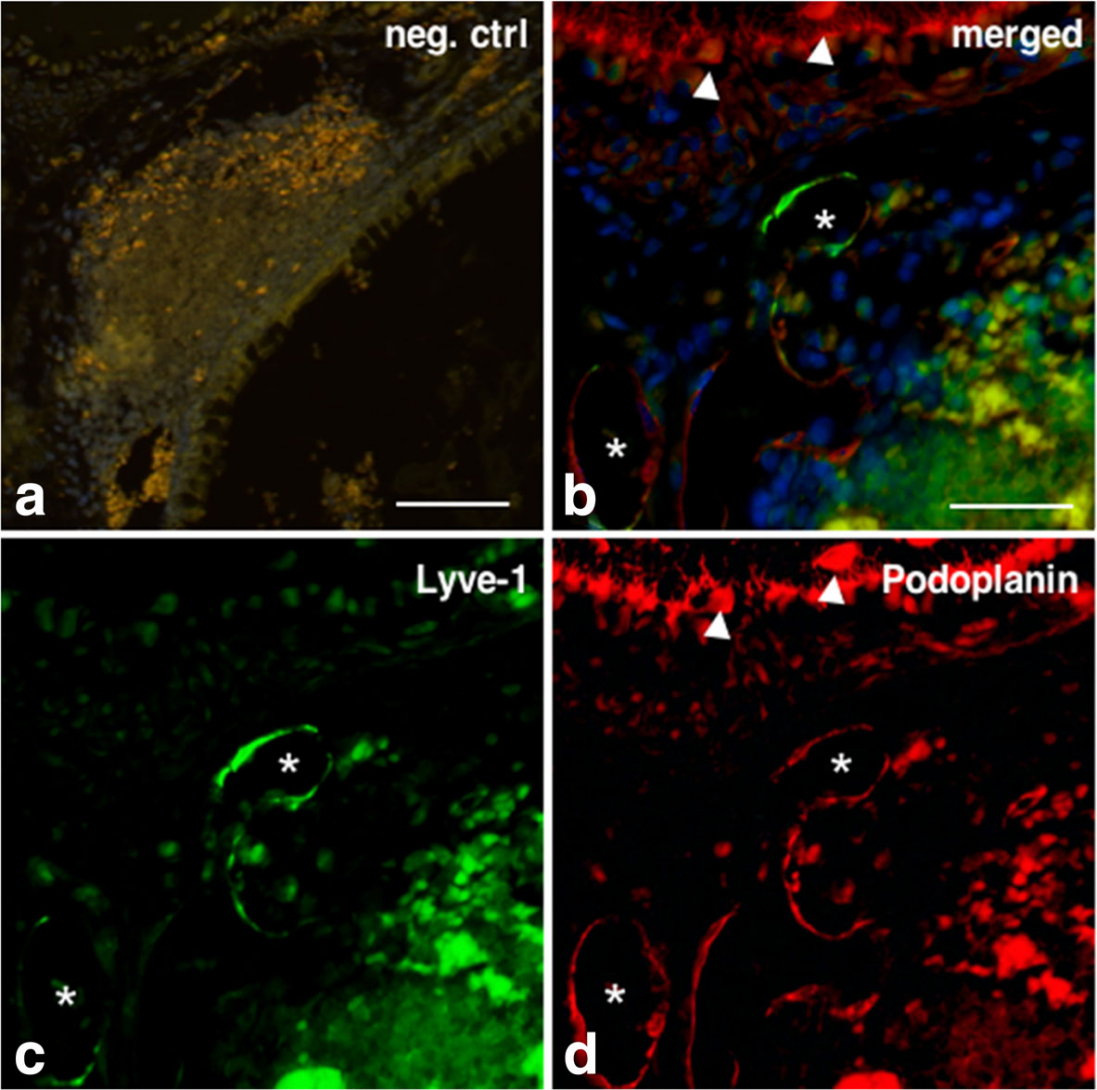
Lymphatics in the NALT-region are Lyve-1^+^ Podoplanin^+^. Shown is an anti-Lyve-1/anti-podoplanin immunofluorescence double-staining on 14-day-old mouse head. Lymph vessels in the NALT-region (indicated by asterisks) are shown to be Lyve-1 (green), as well as Podoplanin (red) positive. Nuclei are stained with Dapi (blue). Arrowheads indicate Podoplanin^+^ osteocytes. Negative control (**a**), green channel (**b**), red channel (**c**), merge (**d**). Magnification 200× (**a**), 400× (**b**-**d**), scale bar represents 50 μm (**a**), 100 μm (**b**-**d**)

Here, we studied heads of 14-day-old and 2-month-old mice with focus on the lymphoid tissues. In consent with previous publications [5, 7], we observed typical lymphoid aggregates in the mucous membrane of the inferior turbinates of 2-month-old mice, that were located frontally of the soft palatine (Fig. 1a), representing the nose-associated lymphoid tissue (NALT). As indicated earlier, we have previously described the lymphatic vascular pattern in the head of 14-day-old mice and showed that the nasal mucous membranes were free of lymphatic vessels, except for the basal parts, which contain lymphatics that form a network with those of the palatine [27]. Accordingly, at the age of 2 months most of the nasal mucous membrane was still free of lymphatics, and only the inferior turbinates with the NALT were characterized by a dense Lyve-1^+^ lymphatic plexus (Fig. 1b, c). More occipitally, we also observed lymphoid aggregates that were located at both sides of the sphenoidal sinus, and again, associated with a network of Lyve-1^+^ lymphatics (Fig. 2). The close spatial relation between NALT and the adjacent lymphatics could already be found in 14-day-old mice (Fig. 3), prior to complete maturation of the NALT. In these younger mice, we observed NALT in the mucous membrane of the inferior turbinates (Fig. 3a, b), as well as its supply with Lyve-1^+^ and Podoplanin^+^ lymphatics (Fig. 3c, d). While Lyve-1 demarcated lymphatic endothelial cells (LECs) highly specifically, Podoplanin could also be found in basal cells of the mucous epithelium, ramified fibroblast-like (dendritic) cells, and osteoblasts. We additionally proved the lymphatic character of the vessels in the NALT region of 14-day-old mice with a Lyve-1/Podoplanin immunofluorescence double staining (Fig. 4).

**Fig. 5:**
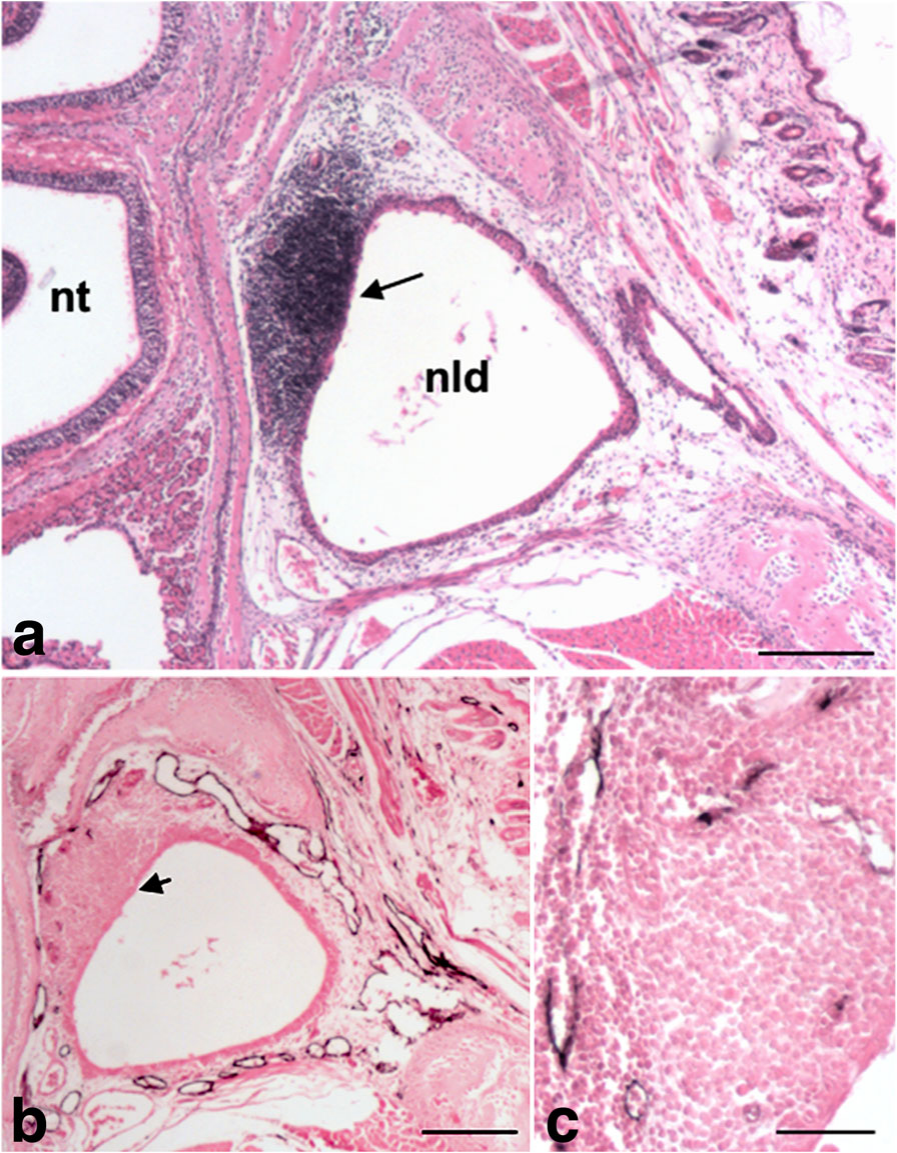
Lymphoid tissue near the nasolacrimal duct and adjacent lymphatics in 14-day-old mice. **a** HE-staining showing the LDALT (arrow) in between the upper nasal turbinates (nt) and the nasolacrimal duct (nld). **b**, **c** Anti-Lyve-1 immunoperoxidase staining showing lymphatics near the LDALT (arrow). 40× magnification, scale bar represents 200 μm (**a**, **b**). 200× magnification, scale bar represents 50 μm (**c**)

**Fig. 6:**
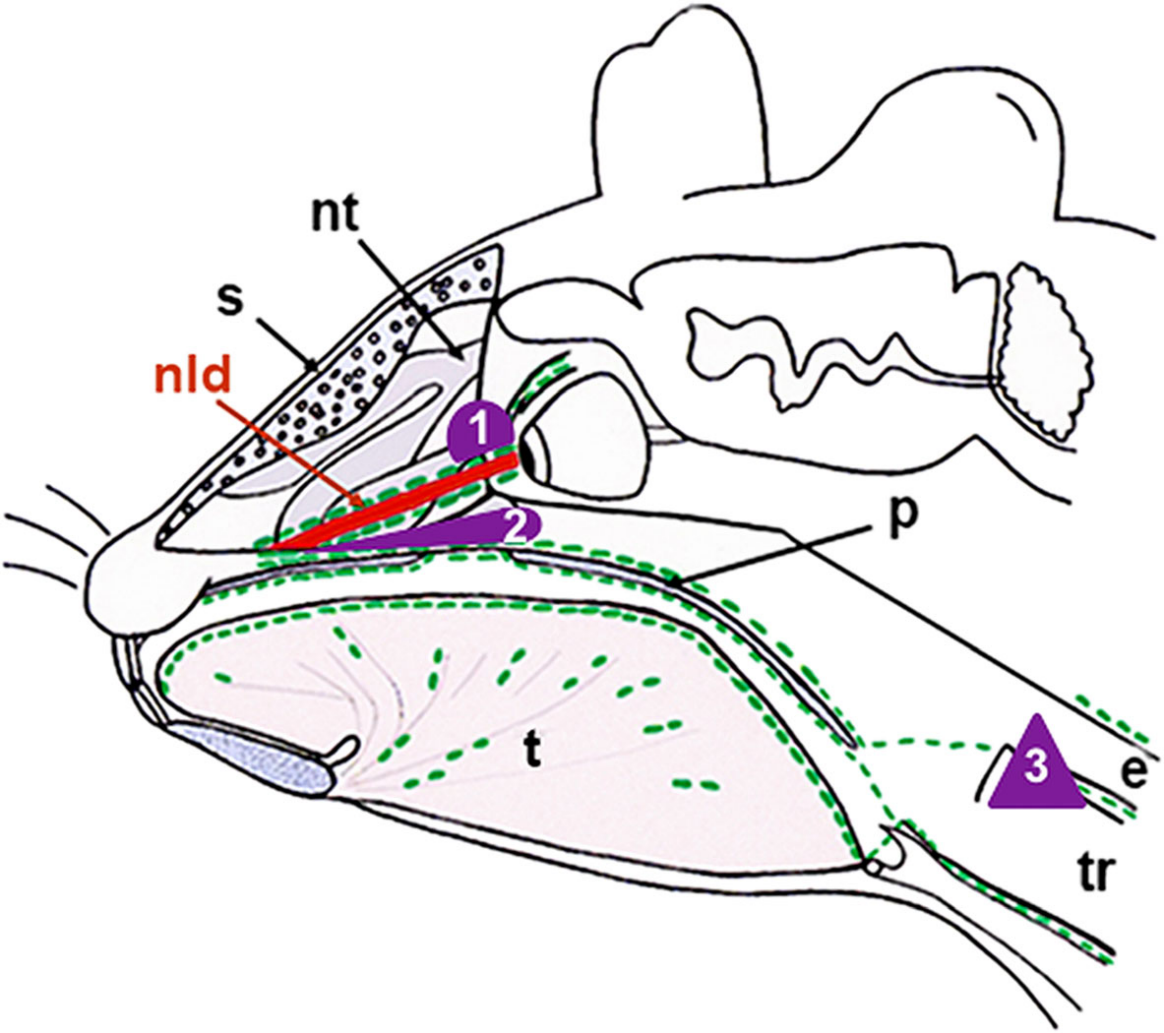
Schematic view of the lymphoid tissues in the mouse head. Positions of the different lymphoid tissues in the mouse head are shown in purple. The LDALT (1) is located near the nasolacrimal duct (nld, red). The NALT (2) is located on the floor of the nasal turbinates (nt) along the palatine (p). Both are densely supplied with lymphatics (green). Along lymphatics of the palatine, antigens of the LDALT and NALT are carried to lymph nodes of the neck region (3). (s, septum; t, tongue, tr, trachea; e, esophagus)

We have previously shown that the periocular lymphatics are connected to those of the inferior turbinates via lymphatic networks that accompany the nasolacrimal duct (NLD) [27]. In 14-day-old mice, we observed lymphoid tissue in close contact to the NLD, near the eye, which has previously been described as lacrimal duct-associated lymphoid tissue (LDALT, also applying to: lacrimal drainage-associated lymphoid tissue) [13]. Again, we observed close association of LDALT with lymphatics, here the ones accompanying the NLD (Fig. 5). In Fig. 6 we schematically show the main lymphoid tissues in the mouse head, that are interconnected with lymph vessels.

## Discussion

The head-and-neck region contains a dense network of lymphatics, which drain into the superficial and deep cervical lymph nodes. In man, lymphoid tissue that is organized as tonsils in Waldeyer’s ring has widely been recognized, however, few studies have dealt with lymphoid tissues in other regions of the head, such as nose-associated lymphoid tissue (NALT) (for review see: [28]), lacrimal duct-associated lymphoid tissue (LDALT) [29, 30], and lymphoid tissue adjacent to the paranasal sinuses [9]. To the best of our knowledge, the association of these lymphoid tissues with the lymphovascular network has not been described yet. Using specific antibodies against LECs in mice, we observed a positive correlation of the developing (14-day-old mice) as well as the mature (2-month-old mice) NALT with the nasal lymphatics, and also a close association of the LDALT with NLD lymphatics. NALT is continued occipitally along both sides of the sphenoidal sinus, referred to as lymphoid tissue adjacent to paranasal sinuses [9], and is again densely supplied by lymphatics. This close connection of lymphoid tissues with the lymphatic vascular network allows immune cells, via efferent lymph, immediate access to the cervical lymph nodes, supporting mucosal immunity. Probably, lymph of the periocular region reaches LDALT via lymphatics sheathing the NLD.

Although NALT and LDALT of humans differ in size and localization from those in mice, most probably due to the presence of tonsils that take over immune responses in the head-and-neck region, these findings might be of clinical relevance, as NALT as well as LDALT have been shown to be involved in different human disorders. In man, lymphoid tissue has been observed along the NLD, and in appr. One third of cases this tissue showed characteristic features of mucosa-associated lymphoid tissue (MALT), whereas in most cases only a diffuse infiltrate of leukocytes was seen in the lamina propria [29]. The examination of 200 lacrimal sacs obtained from patients with chronic dacryocystitis revealed organized lymphatic follicles in 28% of cases and diffuse lymphoid infiltrates in 81% of cases [13]. Notably, these authors noted dilated lacrimal sac lymphatics in chronic dacryocystitis. Lymphoid tissue along the NLD is usually regarded as MALT. However, we have previously shown that the lymphatic vascular network along the NLD is a continuation of the dermal lymphatics [27], consistent with the fact that the NLD epithelium is a derivative of the embryonic epidermis [31]. Lymphoid tissue along the NLD may therefore alternatively be regarded as skin-associated lymphoid tissue (SALT). In this study, we observed lymphoid aggregates at the NLD of mice. As indicated above, these aggregates are in direct contact with lymphatics and may represent the first site for immunological surveillance of pathogens penetrating the conjunctiva. The presence of GP2-expressing M cells in the lacrimal sac and the NLD of mice, as well as the presence of immunoglobulin-producing cells in the lacrimal sac and the NLD of humans are supporting the theory that the NLD and its adjacent lymphatic network play an important role in the mucosal immunity [32, 33].

As rodents do not possess tonsils, it has been assumed that NALT takes over their function [28]. At birth, immature NALT is present in the nasal floor of rats and mice, and already before birth, a few B-cells and some dendritic cells are found beneath the nasal epithelium. However, their position with respect to lymphatics was not studied [7, 34]. In mice, specific B- and T-cell areas, and high endothelial venules (HEVs) are present from day 7 onward, and M-cells can be found in the nasal epithelium [7]. The distribution of lymphocyte subpopulations in NALT is very similar to Peyer’s patches and spleen [28], and therefore, the probability of vaccination via the intranasal route was suggested [35, 36]. Our studies show that, in mice, NALT extends occipitally along lateral aspects of the sphenoidal sinus, and that NALT is connected to lymphatics that accompany the NLD. The question, whether lymphatic vessels connected to NALT, LDALT and NLD are exclusively efferent and thereby drain lymph to the cervical lymph nodes, or whether NALT and LDALT are interconnected by lymphatic vessels along the NLD with efferent and afferent character to improve mucosal immunity, cannot be answered by this study and therefore needs further investigation. A hint for an interconnection is provided by Okada et al., as they could show that LDALT plays a key role in ocular, as well as in nasal immunization and that antigen-specific responses to ocular immunization could be found in both, NALT and LDLAT [37].

As all, except the basal parts of the nasal mucous membrane, are free of lymphatics [27], it can be hypothesized that the presence of lymphatics may have an impact on the development of NALT. Taking into consideration that lymphatics are a key regulator in lymph node development [25] and that the mass of the lymphatic-free areas of the nose do not contain any lymphoid aggregations, we hypothesize that NALT develops by mutual interactions with lymphatics. Additionally, the previously mentioned formation of tertiary lymphoid aggregates in close contact to lymphatics in Crohn’s disease patients supports the theory that the development of lymphoid tissue is linked to the presence of lymphatics [26]. Similar observations have been made in inflammation-induced development of tertiary lymphoid tissue in patients after kidney transplant rejection [38]. However, this hypothesis needs to be further investigated.

## Conclusions

Our study shows the presence of lymphatic vessels in close proximity to NALT and LDALT. Those two lymphatic networks seem to be connected to lymphatics along the NLD. Pathogens that enter the body by crossing the mucosal membranes in the nasal or ocular region can thereby be transported to pharyngeal lymphatic vessels, draining to cervical lymph nodes in the head and neck region. The presence of lymphatic vessels adjacent to the developing as well as to the mature NALT gives a hint that lymphatic vessels might not only be involved in the development of lymph nodes [25], but also in the development of lymphoid tissues in the mouse head.

## Methods

### Animals

For these studies, we used NMRI mice (*n* = 6) at the age of 14 days as well as 2 month after birth, and all experiments were approved by the Lower Saxony state council on animal care (LAVES).

### Tissue preparation, immunohistochemistry, immunofluorescence

Tissue preparation, immunohistochemistry and immunofluorescence were performed as previously described in [23]. For peroxidase Lyve-1 staining of 2-month-old mice and peroxidase podoplanin staining, tyramide-enhanced avidin-biotin immunostaining method was used (Tyramide SuperBoost™ Kit, Invitrogen, Carlsbad, USA; Vectatstain ABC Kit, Vector Laboratories, Eching, Germany).

### Hematoxylin and eosin staining

After deparaffinization and rehydration in xylene and alcohol solutions with descending concentrations, slides were stained in Mayer’s hemalaun solution (Merck, Darmstadt, Germany) for 3 min, shortly differentiated in 0.1% hydrochloric acid and washed under running tap water for 10 min. Afterwards, slides were stained with a 0.5% eosin solution (Eosin G, Carl Roth, Karlsruhe, Germany), shortly washed in Aqua dest. and differentiated using ascending concentrations of alcohol.

## Abbreviations

DCs: Dendritic cells
EDTA: Ethylendiaminetetraacetic acid
GALT: Gut-associated lymphoid tissue
HEVs: High endothelial venules
LDALT: Lacrimal duct-associated lymphoid tissue
LECs: Lymphatic endothelial cells
LTβR: Lymphotoxin β receptor
LTi: Lymphoid tissue inducer
MALT: Mucosa-associated lymphoid tissue
NALT: Nose-associated lymphoid tissue
NLD: Nasolacrimal duct
PBS: Phosphate buffered saline
PFA: Paraformaldehyde
SALT: Skin-associated lymphoid tissue

## References

[1] Miyasaka M, Pabst R, Dudler L, Cooper M, Yamaguchi K (1990). Characterization of lymphatic and venous emigrants from the thymus. Thymus.

[2] Pabst R, Tschernig T (2010). Bronchus-associated lymphoid tissue. An entry site for antigens for successful mucosal vaccination?. Am J Respir Cell Mol Biol.

[3] Pabst R, Ratcliffe MJH (2016). Airway Immune System: microanatomy. Encyclopedia of Immunobiology – volume 3. Elsevier.

[4] Loo S, Chin K (1974). Lymphoid tissue in the nasal mucosa of primates, with particular reference to intraepithelial lymphocytes. J Anat.

[5] Spit B, Hendriksen E, Bruijntjes J, Kuper C (1989). Nasal lymphoid tissue in the rat. Cell Tissue Res.

[6] Kuper C, Koornstra P, Hameleers D, Biewenga J, Spit B, Duijvestijn A, van Breda Vriesman P, Sminia T (1992). The role of nasopharyngeal lymphoid tissue. Immunol Today.

[7] van der Ven I, Sminia T (1993). The devdelopment and structure of mouse nasal-associated lymphoid tissue: an immune- and enzyme-histochemical study. Res Immunol.

[8] Pereira M, Macri N, Creasy D (2011). Evaluation of the rabbit nasal cavity in inhalation studies and a comparison with other common laboratory species and man. Toxicol Pathol.

[9] Debertin A, Tschernig T, Tonjes H, Kleemann W, Troeger H, Pabst R (2003). Nasal-associated lymphoid tissue (NALT): frequency and localization in young children. Clin Exp Immunol.

[10] Kuper C (2006). Histopathology of mucosa-associated lymphoid tissue. Toxicol Pathol.

[11] Bienenstock J, McDermott M (2005). Bronchus- and nasal-associated lymphoid tissues. Immunol Rev.

[12] Paulsen F, Paulsen J, Thale A, Schaudig U, Tillmann B (2002). Organized mucosa-associated lymphoid tissue in human nasolacrimal duct. Adv Exp Med Biol.

[13] Ali M, Mulay K, Pujari A, Naik M (2013). Derangements of lacrimal drainage-associated lymphoid tissue (LDALT) in human chronic Dacryocystitis. Ocul Immunol Inflamm.

[14] Knop E, Knop N (2001). Lacrimal drainage-associated lymphoid tissue (LDALT): a part of the human mucosal immune system. Invest Ophthalmol Vis Sci.

[15] Nagatake T, Fukuyama S, Kim D-Y, Goda K, Igarashi O, Sato S, Nochi T, Sagara H, Yokota Y, Jetten AM, Kaisho T, Akira S, Mimuro H, Sasakawa C, Fukui Y, Fujihashi K, Akiyama T, Inoue J-I, Penninger JM, Kunisawa J, Kiyono H (2009). Id2-, RORγt-, and LTβR-independent initiation of lymphoid organogenesis in ocular immunity. J Exp Med.

[16] Drayton D, Liao S, Mounzer R, Ruddle N (2006). Lymphoid organ development: from ontogeny to neogenesis. Nat Immunol.

[17] Kiyono H, Fukuyama S (2004). Nalt- versus peyer's-patch-mediated mucosal immunity. Nat Rev Immunol.

[18] Mebius R (2003). Organogenesis of lymphoid tissue. Nat Rev Immunol.

[19] van de Pavert S, Mebius R (2010). New insights into the development of lymphoid tissues. Nat Rev Immunol.

[20] Krege J, Seth S, Hardtke S, Davalos-Misslitz AC, Förster R (2009). Antigen-dependent rescue of nose-associated lymphoid tissue (NALT) development independent of LTbetaR and CXCR5 signaling. Eur J Immunol.

[21] Fukuyama S, Hiroi T, Yokota Y, Rennert P, Yanagita M, Kinoshita N, Terawaki S, Shikina T, Yamamoto M, Kurono Y, Kiyono H (2002). Initiation of NALT organogenesis is independent of the IL-7R, LTbetaR, and NIK signaling pathways but requires the Id2 gene and CD(−)CD4(+)CD45(+) cells. Immunity.

[22] Harmsen A, Kusser K, Hartson L, Tighe M, Sunshine M, Sedgwick J, Choi Y, Littman D, Randall T (2002). Cutting edge: organogenesis of nasal-associated lymphoid tissue (NALT) occurs independently of Lymphotoxin- (LT) and retinoic acid receptor-related orphan receptor-, but the organization of NALT is LT dependent. J Immunol.

[23] Fukuyama S, Nagatake T, Kim D, Takamura K, Park E, Kaisho T, Tanaka N, Kurono Y, Kiyono H (2006). Cutting edge: uniqueness of lymphoid chemokine requirement for the initiation and maturation of Nasopharynx-associated lymphoid tissue organogenesis. J Immunol.

[24] Rangel-Moreno J, Moyron-Quiroz J, Kusser K, Hartson L, Nakano H, Randall T (2005). Role of CXC chemokine ligand 13, CC chemokine ligand (CCL) 19, and CCL21 in the organization and function of nasal-associated lymphoid tissue. J Immunol.

[25] Onder L, Mörbe U, Pikor N, Novkovic M, Cheng HW, Hehlgans T, Pfeffer K, Becher B, Waismann A, Rülicke T, Gommermann J, Mueller CG, Sawa S, Scandella E, Ludewig B (2017). Lymphatic endothelial cells control initiation of lymph node organogenesis. Immunity.

[26] Randolph G, Bala S, Rahier J, Johnson M, Wang P, Nalbantoglu I, Dubuquoy L, Chau A, Pariente B, Kartheuser A, Zinselmeyer B, Colombel J (2016). Lymphoid aggregates remodel lymphatic collecting vessels that serve mesenteric lymph nodes in Crohn disease. Am J Pathol.

[27] Lohrberg M, Wilting J (2016). The lymphatic vascular system of the mouse head. Cell Tissue Res.

[28] Pabst R (2015). Mucosal vaccination by the intranasal route. Nose-associated lymphoid tissue (NALT) – structure, function and species differences. Vaccine.

[29] Paulsen FP, Schaudig U, Maune S, Thale AB (2003). Loss of tear duct-associated lymphoid tissue in association with the scarring of symptomatic darcryostenosis. Ophthalmology.

[30] Zhang T, Wang J, Wang L, Shan Y (2004). Morphological study of nasolacrimal duct mucosa-associated lymphoid tissue. Chinese.

[31] Moore K, Persaud T, Torchia M (2015). The developing human: clinically oriented embryology.

[32] Sirigu P, Maxia C, Puxeddu R, Zucca I, Piras F, Perra MT (2000). The presence of a local immune system in the upper blind and lower part of the human nasolacrimal duct. Arch Histol Cytol.

[33] Kimura S, Kishimoto A, Mutoh M, Takahashi-Iwanaga H, Iwanaga T (2015). GP2-expressing cells in the conjunctiva and tear ducts of mice: identification of a novel type of cells in the squamous stratified epithelium. Biomed Res.

[34] Hameleers DM, van der Ende M, Biewenga J, Sminia T (1989). An immunohistochemical study on the postnatal development of rat nasal-associated lymphoid tissue. Res Immunol.

[35] Kim DY, Sato A, Fukuyama S, Sagara H, Nagatake T, Kong IG, Goda K, Nochi T, Kunisawa J, Sato S, Yokota Y, Lee CH, Kiyono H (2011). The airway antigen sampling system: respiratory M cells as an alternative gateway for inhaled antigens. J Immunol.

[36] Jahnsen FL, Gran E, Haye R, Brandtzaeg P (2004). Human nasal mucosa contains antigen-presenting cells of strikingly different functional phenotypes. Respir Cell Mol Biol.

[37] Okada K, Yamasoba T, Kiyono H, Harabuchi Y (2011). Cranifacial mucosal immune system: importance of its unique organogenesis and function in the development of a mucosal vaccine. Recent Advances in Tonsils and Mucosal Barriers of the Upper Airways Basel: Karger.

[38] Kerjaschki D, Huttary N, Raab I, Regele H, Bojarski-Nagy K, Bartel G, Kröber SM, Greinix H, Rosenmaier A, Karlhofer F, Wick N, Mazal PR (2006). Lymphatic endothelial progenitor cells contribute to de novo lymphangiogenesis in human renal transplants. Nat Med.

